# Phenotypic alterations in type II alveolar epithelial cells in CD4^+ ^T cell mediated lung inflammation

**DOI:** 10.1186/1465-9921-8-47

**Published:** 2007-07-04

**Authors:** Marcus Gereke, Lothar Gröbe, Silvia Prettin, Michael Kasper, Stefanie Deppenmeier, Achim D Gruber, Richard I Enelow, Jan Buer, Dunja Bruder

**Affiliations:** 1 Immune Regulation Group, Helmholtz Centre for Infection Research, Braunschweig, Germany; 2 Department of Mucosal Immunity, Helmholtz Centre for Infection Research, Braunschweig, Germany; 3 Institute of Anatomy, Medical Faculty Carl Gustav Carus, Dresden University of Technology, Dresden, Germany; 4 Department of Veterinary Pathology, Free University Berlin, Berlin, Germany; 5 Departments of Medicine, and Microbiology/Immunology, Dartmouth Medical School, Lebanon, NH, USA; 6 Department of Medical Microbiology, University Hospital Essen, Essen, Germany

## Abstract

**Background:**

Although the contribution of alveolar type II epithelial cell (AEC II) activities in various aspects of respiratory immune regulation has become increasingly appreciated, our understanding of the contribution of AEC II transcriptosome in immunopathologic lung injury remains poorly understood. We have previously established a mouse model for chronic T cell-mediated pulmonary inflammation in which influenza hemagglutinin (HA) is expressed as a transgene in AEC II, in mice expressing a transgenic T cell receptor specific for a class II-restricted epitope of HA. Pulmonary inflammation in these mice occurs as a result of CD4^+ ^T cell recognition of alveolar antigen. This model was utilized to assess the profile of inflammatory mediators expressed by alveolar epithelial target cells triggered by antigen-specific recognition in CD4^+ ^T cell-mediated lung inflammation.

**Methods:**

We established a method that allows the flow cytometric negative selection and isolation of primary AEC II of high viability and purity. Genome wide transcriptional profiling was performed on mRNA isolated from AEC II isolated from healthy mice and from mice with acute and chronic CD4^+ ^T cell-mediated pulmonary inflammation.

**Results:**

T cell-mediated inflammation was associated with expression of a broad array of cytokine and chemokine genes by AEC II cell, indicating a potential contribution of epithelial-derived chemoattractants to the inflammatory cell parenchymal infiltration. Morphologically, there was an increase in the size of activated epithelial cells, and on the molecular level, comparative transcriptome analyses of AEC II from inflamed versus normal lungs provide a detailed characterization of the specific inflammatory genes expressed in AEC II induced in the context of CD4^+ ^T cell-mediated pneumonitis.

**Conclusion:**

An important contribution of AEC II gene expression to the orchestration and regulation of interstitial pneumonitis is suggested by the panoply of inflammatory genes expressed by this cell population, and this may provide insight into the molecular pathogenesis of pulmonary inflammatory states. CD4^+ ^T cell recognition of antigen presented by AEC II cells appears to be a potent trigger for activation of the alveolar cell inflammatory transcriptosome.

## Background

The epithelium constitutes the interface between the internal milieu and the external environment, and the respiratory epithelium is the initial point of contact for respiratory viruses, airborne allergens and environmental pollutants [1]. The major function of the respiratory epithelium was at one time felt to be primarily that of a physical barrier, but recent studies clearly indicate that its cells are metabolically very active with the capacity to modulate a variety of inflammatory processes through the action of an array of receptor-mediated events. Upon activation, epithelial cells have the capacity to produce a number of pro-inflammatory or regulatory mediators, including arachidonic acid products, nitric oxide, endothelin-1, transforming growth factor (TGF)-β, tumour necrosis factor (TNF)-α, and cytokines such as interleukin (IL)-1, IL-6 and IL-8 [2].

Alveolar type II epithelial cells (AEC II, granular pneumocyte, type II pneumocyte, giant corner cell) represent a highly specialized subpopulation of the respiratory epithelium. AEC II consist of about 15% of the distal lung cells and occupy 5% of the alveolar surface [3]. They perform a variety of important functions within the lung, including regulation of surfactant metabolism, ion transport and alveolar repair in response to injury [4-7]. AEC II synthesize and secrete lung surfactant, a protein-lipid complex and surface-active material [8]. Ultrastructural criteria used to identify alveolar type II epithelial cells are the presence of lamellar bodies, apical microvilli and specific junctional proteins. AEC II also maintain the integrity of alveolar epithelium by proliferation (and differentiation to type I cells) in response to injury, and tightly regulate alveolar fluid by a variety of mechanisms.

AEC II express a number of molecules necessary for the transduction as well as the generation of signals involved in cell-cell as well as in cell-matrix interactions. Cell-cell interactions may be direct via contact of tight junction proteins, or indirect via secreted and diffusible signals [9]. Consequently, AEC II have been described as integrative units of the alveolus [10]. Interactions of AEC II with leukocytes have also been the subject of intense investigation and there is evidence supporting a role of AEC II in accessory function in T lymphocyte activation [11,12]. Moreover AEC II chemokine expression is induced upon antigen-specific CD8^+ ^T cell recognition and plays a critical role in the perpetuation of experimental interstitial pneumonia [13,14].

In order to study the pathophysiology of chronic T cell-mediated lung injury, we established a novel model in which a model antigen (influenza A/PR8/34 HA) is expressed under the control of the SP-C promoter, resulting in AEC II cell-specific expression and bred these animals with mice expressing a transgenic T cell receptor, specific for a class II-restricted epitope of HA, leading to a chronic interstitial pneumonitis [15]. Initial characterization of these mice focussed on self-antigen specific T cell function and revealed the induction of peripheral T cell tolerance at the site of inflammation. In this study we demonstrate altered AEC II cell morphology in mice with CD4^+ ^T cell-mediated pulmonary inflammation suggesting a state of activation that we wanted to explore at a molecular level. As such, we established a method to isolate highly pure primary AEC II for the purpose of performing *ex vivo *expression profiling in the context of acute and chronic interstitial pneumonitis. An important role of AEC II gene expression in the orchestration of inflammatory infiltration of the lung parenchyma is suggested by a wide array of inflammatory genes and chemoattractants expressed upon CD4^+ ^T cell recognition of antigen presented by the AEC II cells, and this model may prove extremely useful in dissecting the mechanisms involved in the perpetuation of chronic autoimmune pulmonary processes.

## Methods

### Mice and antibodies

BALB/c mice were obtained from Harlan (Borchen, Germany). TCR-HA transgenic mice expressing a TCR aβ specific for the I-E^d^-restricted HA-peptide 110–120 from A/PR8/34 HA have been described previously [16]. SPC-HA mice expressing the influenza A/PR8/34 HA under the transcriptional control of the human surfactant protein C (SP-C) promoter specifically in AEC II have been described elsewhere [15]. Mice were bred in the animal facility at the Helmholtz Centre for Infection Research and were kept under SPF conditions. All mice were routinely monitored for the absence of bacterial, viral, parasitic and fungal infections. Mice aged 10 to 20 weeks were used for experiments which were all performed according to national and institutional guidelines. The monoclonal antibody 6.5 (anti-TCR-HA) was purified from hybridoma supernatants by protein G affinity chromatography. The antibodies a-CD45 (30-F11), a-CD16/CD32 (2.4G2), a-CD11b (M1/70) and a-F4/80 were obtained from BD Biosciences and used either unconjugated or as phycoerythrin (PE) conjugates. As secondary polyclonal goat a-rat IgM/IgG/IgA was used as phycoerythrin (PE) conjugate. For specific staining of sorted AEC II, the lectin Maclura pomifera agglutinin was used. Intracellular staining for IFN-γ and IL-2 was performed using the antibodies a-IFN-γ (XMG1.2) and a-IL-2 (JES6-5H4) from BD Biosciences, according to the manufacturer's protocol.

### Adoptive transfer of HA-specific CD4^+ ^T cells

Naïve CD4^+ ^T cells from the spleens of TCR-HA mice were isolated by negative selection by AutoMACS using the CD4^+ ^T cell isolation kit from Miltenyi Biotec (Bergisch Gladbach, Germany), followed by i.v. injection of 1 × 10^6 ^antigen-specific CD4^+ ^T cells into SPC-HA transgenic mice. At various time points after transfer, animals were sacrificed and lungs perfused with PBS prior to excision. The lungs were sectioning for histological analysis and quantitative morphometry or were used for isolation of AEC II cells, or infiltrating lymphocytes, as described below.

### Isolation of lymphocytes from the lung

Perfused lungs were excised and finely minced on ice, followed by a 60–90 minutes digestion at 37°C with collagenase/dispase (0,2 mg/ml each) in IMDM/5% FCS in the presence of 25 μg/ml DNase. To improve tissue disintegration, lungs were pipeted every 5 min using a Pasteur pipet. EDTA was added to a final concentration of 5 mM followed by an additional 5 min incubation at 37°C. Cells were passed through a 70 μm cell strainer, washed, and lymphocytes isolated by density centrifugation.

### Isolation of alveolar type II epithelial cells

Primary AEC II were prepared using a modified protocol of a previously published method [17]. Briefly, mice were anesthetized and exsanguinated by serving the inferior vena cava and left renal artery. The tracheae was exposed and cannulated and lungs were perfused with 10 to 20 ml sterile phosphate buffered saline via the pulmonary artery until visually free of blood. 2 ml dispase (BD Biosciences, Heidelberg, Germany) was instilled into lungs via the tracheal catheter followed by instillation of 500 μl 1% low-melt agarose prior warmed to 45°C. Instilled lungs were immediately covered with ice and incubated for 2 min to gel the agarose. Lungs were removed, placed in a culture tube containing an additional 1 ml of dispase and incubated for 45 min at room temperature. The lungs were then transferred to a culture dish and 7 ml serum free DMEM + 25 mM HEPES (GIBCO, Eggenstein, Germany) containing 100U/ml DNase I (Sigma, Hannover, Germany) was added. The tissue was gently teased away from the airways using forceps and lungs were carefully dissociated before agitating the tissue for 10 min on a shaker. Crude cell suspensions were sequentially filtered through nylon gauze (100 μm, 45 μm, 30 μm) followed by centrifugation (12 min, 130 × g) to pellet the cells. For fluorescence activated cell sorting of alveolar type II epithelial cells, cells were washed with serum free DMEM + 25 mM HEPES and subsequently labelled with anti-CD45, anti-CD32/CD16, anti-CD11b and anti-F4/80 antibodies and PE-conjugated goat anti rat-IgG as secondary antibody. After staining the cell suspension was washed with PBS containing 2% fetal calf serum and 2 mM EDTA and subjected to one-step cell sorting using a MoFlow cell sorter (Cytomation, Fort Collins, CO). Granular alveolar type II epithelial cells were identified as SSC^high ^population. PE (CD45/CD32/CD16/CD11b/F4/80)-positive cells were excited by an argon ion laser emitted at the wavelength of 488 nm and the fluorescence was collected after a 580/±30 nm band-pass filter. A two parameter sorting window (side light scattering and PE fluorescent intensity) was used to identify the PE-negative, side scatter high AEC II population. Cells were sorted through a flow chamber with a 100 μm nozzle tip under 25 psi sheath fluid pressure. Using this protocol a purity of 97–99% and viability of 90% was obtained. Isolated cells were either used for immunofluorescence staining or RNA preparation.

### Histology

Lungs were perfused and fixed with neutral buffered formalin, embedded in paraffin, sectioned and stained with hematoxylin and eosin (H&E).

### Immunofluorescence

For immunofluorescence staining sorted AEC II were mounted onto glass cover slips with a density of 2 × 10^5 ^cells using a cytospin apparatus and were fixed with methanol-acetone (1:1) mixture at -20°C for 5 min. Rabbit anti SP-A, SP-B, pro-SPC and SP-D antibodies (Chemicon Europe, Hampshire, UK) were all diluted 1:100 and incubated with the fixed cells overnight at 4°C. A secondary FITC conjugated goat anti-rabbit IgG (Dianova, Hamburg, Germany) was used with a dilution of 1:80 and stained for 30 min at 37°C. All washing steps were performed in PBS and stained cells were embedded in glycerol-PBS before microscopic examination.

### DNA microarray hybridization and analysis

Total RNA from AEC II sorted from the lung of either healthy SPC-HA or diseased SPC-HA/TCR-HA mice was isolated using the RNAeasy kit (Qiagen, Hilden, Germany). Quality and integrity of total RNA isolated from 2 × 10^5 ^sorted AEC II cells was assessed by running all samples on an Agilent Technologies 2100 Bioanalyser (Agilent Technologies, Waldbronn, Germany). For RNA amplification the first round was performed in accordance with an Affymetrix protocol without biotinylated nucleotides, using the Promega P1300 RiboMax Kit (Promega, Mannheim, Germany) for T7 amplification. For the second round of amplification the precipitated and purified RNA was converted to cDNA primed with random hexamers (Pharmacia, Freiburg, Germany). Second strand synthesis and probe amplification were done as in the first round, with two exceptions: incubation with RNase H preceded the first strand synthesis to digest the aRNA; and the T7T23V oligonucleotide was used for initiation of the second strand synthesis. 12.5 μg biotinylated cRNA preparation was fragmented and placed in a hybridization cocktail containing four biotinylated hybridization controls (BioB, BioC, BioD, and Cre) as recommended by the manufacturer. Samples were hybridized to an identical lot of either Affymetrix MOE430A or MOE4302.0 chips for 16 hours. After hybridization, GeneChips were washed, stained with streptavidin-PE and read using an Affymetrix GeneChip fluidic station scanner. Analysis was done with gene expression software (GeneChip, MicroDB, and Data Mining Tool, all Affymetrix).

### Real-time RT-PCR

Total RNA was prepared from sorted AEC II cells using the RNeasy kit (Qiagen, Hilden, Germany) and cDNA synthesis was done using Superscript II Reverse Transcriptase, Oligo dT and random hexamer primers (Invitrogen). Quantitative Real-time RT-PCR was performed on an ABI PRISM cycler (Applied Biosystems) using a SYBR Green PCR kit from Stratagene and specific primers optimized to amplify 90–250 bp fragments from the various genes analyzed. A threshold was set in the linear part of the amplification curve and the number of cycles needed to reach this was calculated for every gene. Relative mRNA levels were determined by using included standard curves for each individual gene and further normalization to RPS9. Melting curves were used to establish the purity of the amplified band.

## Results

### CD4^+ ^T cell recognition of epithelial antigen results in interstitial inflammation accompanied by AEC II hypertrophy

**Figure 1:**
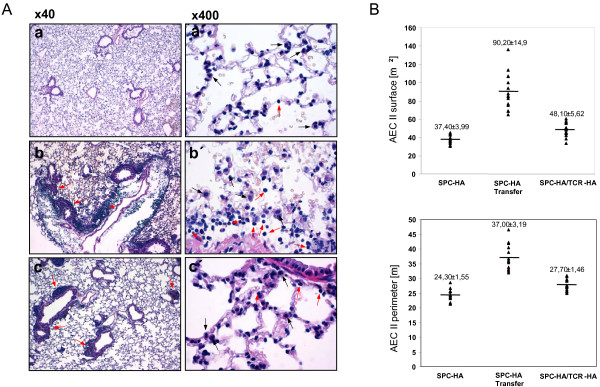
**CD4^+ ^T cell recognition of alveolar epithelial antigen results in airway inflammation and AEC II hypertrophy**. (A) Histological examination of lungs from healthy SPC-HA (a and a'), SPC-HA six days after adoptive transfer of HA-specific CD4^+ ^T cells (b, b') and SPC-HA/TCR-HA double transgenic mice (c, c'). Lung sections were stained with H&E. Black arrows indicate AEC II, red arrows indicate lymphocytes. No lesions were detectable in the lung of SPC-HA mice. Specifically, type II pneumocytes were completely unchanged (a, a'). A moderate, perivascular and peribronchiolar infiltration with mature lymphocytes was detected in the lung of SPC-HA mice after transfer with HA-specific CD4^+ ^T cells. Adjacent to these infiltrations, a slight connective tissue edema and a mild infiltration with neutrophils were observed. Type II pneumocytes in the vicinity of the lymphocytic infiltrations were moderately hypertrophic. A few alveolar macrophages were present in the alveoli (b, b'). Moderate, multifocal, perivascular and peribronchiolar infiltrations with lymphocytes were present in the lung of SPC-HA/TCR-HA double transgenic mice. Type II pneumocytes close to the lymphocytic infiltrations were mildly activated and hypertrophic (c, c'). **(B) **Histological results were corroborated morphometrically by measuring AEC II surface and perimeter to quantify the degree of cellular hypertrophy (n = 15, 3 mice with 5 AEC II per mouse; ± standard deviation). AEC II surface: SPC-HA vs SPC-HA Transfer: P < 0,001), SPC-HA vs SPC-HA/TCR-HA (P < 0,0001), SPC-HA transfer vs SPC-HA/TCR-HA (P < 0,0001). AEC II perimeter: SPC-HA vs SPC-HA Transfer: P < 0,001), SPC-HA vs SPC-HA/TCR-HA (P < 0,001), SPC-HA transfer vs SPC-HA/TCR-HA (P < 0,001). All Student's t-test.

**Figure 2:**
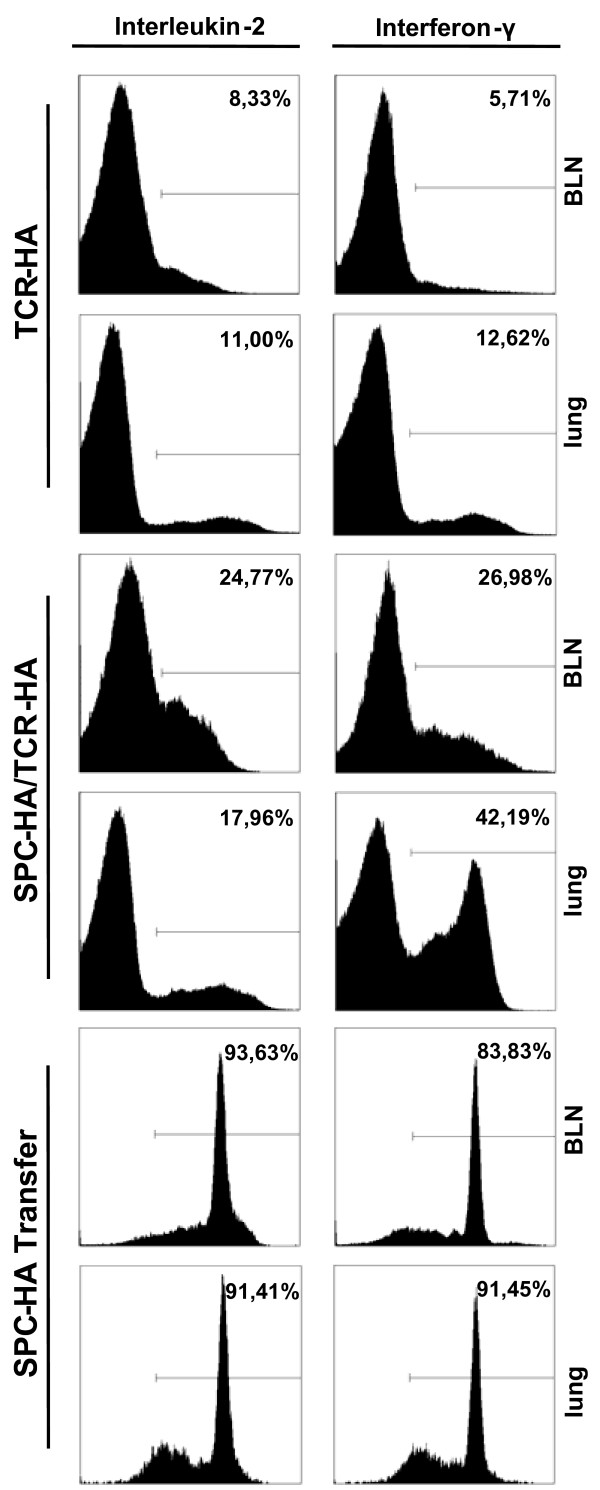
**Intracellular cytokine staining in CD4^+ ^T cells**. CD4^+ ^T cells from the lung or bronchial lymph nodes (BLN) from either TCR-HA control mice, SPC-HA/TCR-HA double transgenic mice or SPC-HA mice adoptively transferred with HA-specific CD4^+ ^T cells were analyzed by FACS for the expression of interleukin 2 and interferon γ.

We have previously shown that HA expressed by AEC II in SPC-HA transgenic mice results in presentation of a MHC class II-restricted epitope to CD4^+ ^T cells and lung pathology [15]. Immunopathology, characterized by massive lymphocytic infiltration of interalveolar septa, was observed both in SPC-HA mice that were adoptively transferred with HA-specific CD4^+ ^T cells as well as in SPC-HA mice that were crossed with TCR-HA mice to establish autoimmune conditions (Figure 1A). Interestingly, the histologic appearance of AEC II cells in acutely inflamed lungs revealed that they were in close contact with lymphocytes and displayed an activated phenotype with cellular hypertrophy, characterized by significantly increased AEC II surface area and perimeter. This was most prominent during acute inflammation (i.e. shortly after adoptive transfer) and was less evident in the chronic inflammatory state in adult SPC-HA/TCR-HA mice (Figure 1B and [15]). Accordingly, CD4^+ ^T cells isolated from the lung of SPC-HA mice shortly after adoptive transfer produced elevated levels of the pro-inflammatory cytokines IL-2 and IFN-γ compared with T cells isolated from the lungs of SPC-HA/TCR-HA mice at 16–20 weeks of age (Figure 2).

### Isolation of type II alveolar epithelial cells

**Figure 3:**
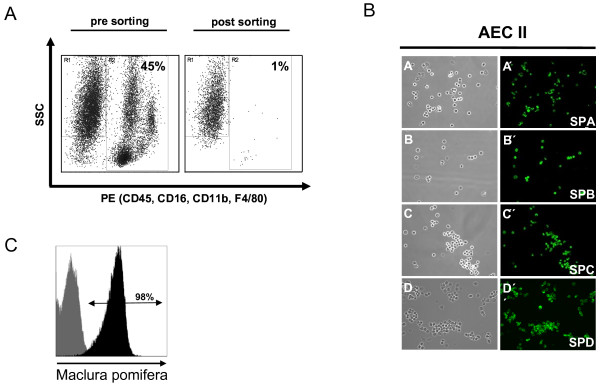
**Purification of alveolar type II epithelial cells by fluorescence-activated cell sorting**. (A) Cell suspension obtained by enzymatic tissue disintegration and subsequent sequential filtration was labelled with antibodies to CD45, CD16, CD32, CD11b, and F4/80. Antibody negative AEC II were further distinguished from other cells by size and granularity. Reanalysis of sorted cells demonstrated an extremely low frequency of contaminating hematopoetic cells. (B) Sorted cells express surfactant proteins A, B, C and D. Cytospins of sorted AEC II cells were stained for the surfactant proteins A, B, C and D. Almost all cells were found to be positive for all four surfactant proteins. A, B, C and D represent phase contrast microscopy, A', B', C', and D' represent immunohistochemical stainings for the corresponding surfactant protein. (C) Staining of sorted AEC II with Maclura pomifera lectin revealed high purity of isolated cells. Black histogram indicates staining with the lectin, grey histogram indicates unstained cells.

To assess the contribution of AEC II to the orchestration and progression of T cell-mediated interstitial pneumonitis in more detail, we established a protocol for isolation of AEC II from the murine lung entirely by negative selection. Enzymatic digestion and antibody staining, followed by sorting of SSC^high ^and CD45/CD32/CD16/CD11/F4/80^negative ^cells, resulted in highly pure and viable AEC II cells, as indicated by surfactant protein (SP)-A, -B, -C and -D expression (Figure 3A,B). Identity of sorted cells as type II pneumocytes was further confirmed by staining with the lectin Maclura pomifera agglutinin, that specifically binds to a 185 kDa glycoprotein on AEC II but not on alveolar type I epithelial cells (AEC I) [18]. As depicted in Figure 3C, essentially all cells stained positive with the lectin, demonstrating high purity of AEC II cells obtained by negative selection cell sorting.

### Global changes in AEC II gene expression following CD4^+ ^T cell recognition of alveolar antigen

To characterize alterations in the transcriptional program of alveolar epithelial cells in the context of T cell-mediated interstitial pneumonitis, we performed gene expression arrays on primary AEC II cells isolated from the lung of either healthy SPC-HA mice or 16–20 week old SPC-HA/TCR-HA mice with autoimmune lung inflammation. As previously mentioned, SPC-HA/TCR-HA mice develop a spontaneous pneumonitis due to the concomitant expression of the neo-self antigen influenza HA in AEC II and a transgenic TCR specifically recognizing an I-E^d^-restricted epitope from this particular antigen [15]. Thus, lung inflammation occurs as a consequence of CD4^+ ^T cell recognition of a single alveolar epithelial "self antigen".

For gene expression analysis, RNA prepared from AEC II was subjected to differential gene expression analysis using oligonucleotide microarrays. An important advantage of this technology is that every analyzed gene is represented by sixteen independent probe pairs which together establish the basis for statistical evaluations of the respective signals. Therefore, only the genes that are reproducibly regulated are included in the analysis. For each gene fulfilling these criteria, the average fold change in expression for AEC II from the inflamed lung of SPC-HA/TCR-HA and healthy lung of SPC-HA mice was calculated and the ratio was depicted on a base-2 logarithmic scale. To establish the basal expression level of analyzed genes in AEC II under non-pathologic conditions, an alignment of AEC II derived from the healthy and inflamed lungs was also performed, in duplicate arrays. The number of "present calls" (42.1 to 44.7%) as calculated by the statistical detection algorithm of Affymetrix was similar to data obtained from analysis of other types of cells, e.g. T lymphocytes isolated by cell sorting [15].

**Figure 4:**
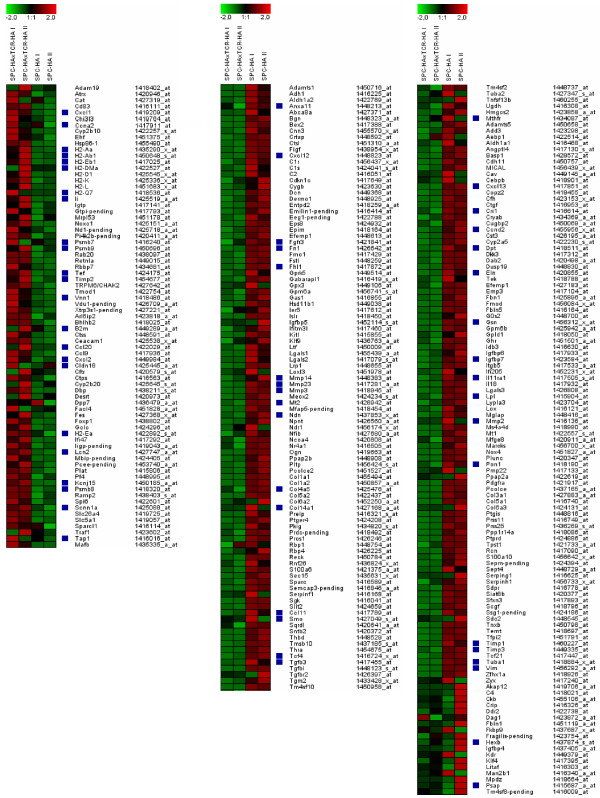
**Heat map including genes differentially expressed in AEC II cells isolated from lungs of diseased SPC-HA/TCR-HA as well as healthy SPC-HA mice**. Red indicates induction of gene expression, green indicates repression (+2: bright red; -2: bright green). Black indicates no changes. Blue squares indicate genes further highlighted in Table 1. Genes were considered to be regulated whose expression was at least twofold increased or decreased.

The purity and integrity of isolated AEC II was examined using basal gene expression levels of selected genes in AEC II isolated from the lungs of healthy SPC-HA mice. Consistent with results obtained by immunofluorescence microscopy (Figure 3), sorted AEC II cells showed high mRNA expression levels for SP-A, SP-B, SP-C and SP-D (data not shown). Comparison of expression profiles of AEC II cells from healthy and inflamed lungs revealed 322 genes that exhibited more than a two-fold expression change. Among these, 288 encode proteins of known or putative function (depicted in Figure 4), and the remaining 34 genes are currently described as expressed sequence tags (ESTs) or encoding unknown proteins. The full list of differentially expressed genes is accessible online at [19].

Regulated genes were grouped into 11 functional classes by their putative functions (Table 1). Among the genes most significantly regulated in association with interstitial inflammation were genes encoding the chemokine CCL20, matrix metalloproteinases 2 and 3, and tissue inhibitor of metalloproteinase 1. Also, strong down-regulation of expression of several genes associated with cell adhesion, including procollagen type XIV, alpha 1, fibronectin 1 and dermatopontin, was observed in AEC II cells isolated from the inflamed lung. Interestingly, whereas many genes involved in signal transduction (such as lipoprotein lipase, prosaponin and metallothionein 2) and cytoskeletal function (such as gelsolin and vimentin) were down-regulated, genes involved in antigen processing and presentation, such as MHC class II subunits, proteasome subunits and beta-2 microglobulin exhibited elevated expression in the inflamed lung. These genes along with other potentially interesting genes differentially expressed in AEC II cells isolated from the inflamed lung, are listed in Table 1.

**Figure 5:**
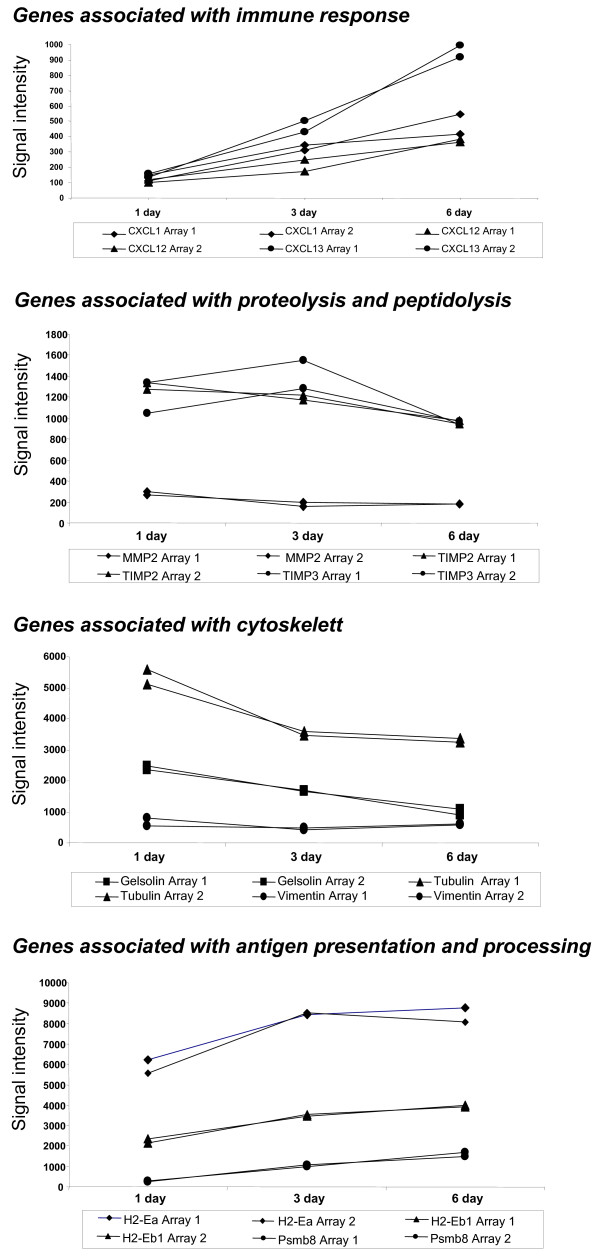
**Time course of gene expression in AEC II after adoptive CD4^+ ^T cell transfer into SPC-HA mice**. AEC II cells were isolated from the lung of SPC-HA mice one (n = 3), three (n = 3) and six (n = 3) days after adoptive transfer of HA-specific CD4^+ ^T cells. Cells were subjected to microarray analysis and the level of gene expression over time is depicted for selected genes. Data obtained from two different experiments are represented.

**Figure 6:**
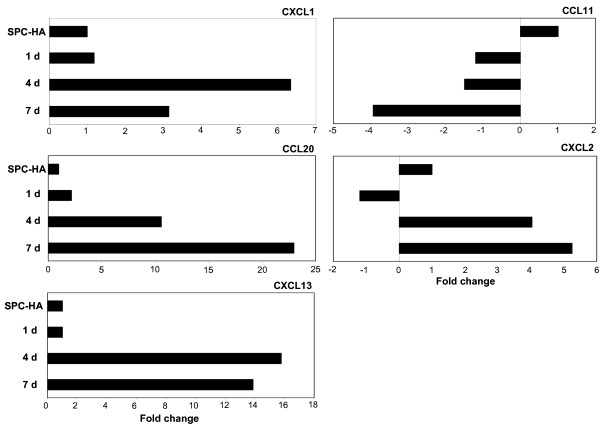
**Chemokine expression in AEC II after adoptive CD4^+ ^T cell transfer into SPC-HA mice**. AEC II cells were isolated from the lung of SPC-HA mice one (n = 3), three (n = 3) and six (n = 3) days after adoptive transfer of HA-specific CD4^+ ^T cells. Cells were subjected to quantitative real-time RT-PCR analyses. mRNA expression levels of CXCL1, CCL20, CXCL13, CCL11, CXCL2, and RPS9 (as internal control) were analyzed in real-time RT-PCR assays. Relative mRNA amounts were normalized with respect to expression levels in AEC II cells isolated from SPC-HA mice not receiving CD4^+ ^T cell transfer (fold change = 1).

The morphology of AEC II differed considerably between SPC-HA mice that were adoptively transferred with HA-specific CD4^+ ^T cells, and analyzed acutely, compared with those crossed to TCR-HA mice, and analyzed during a chronic phase (Figure 1), suggesting a more pronounced pro-inflammatory participation of AEC II during the acute phase of inflammation. We therefore extended the gene expression profiling to AEC II isolated 1, 3 or 6 days after transfer, in order to examine the early activation events in greater detail. Selected genes including genes associated with immune responses, proteolysis and peptidolysis, cytoskeletal function, and antigen presentation and processing were analyzed for changes in expression over time (Figure 5). In addition, AEC II expression of selected chemokines in the acute phase of lung inflammation was further validated by quantitative real-time RT-PCR analyses (Figure 6). Interestingly, for the majority of genes analyzed the changes in the expression level observed acutely mirrored the chronic changes observed in AEC II isolated from the lung of SPC-HA/TCR-HA mice at 16–20 weeks. Thus, the alterations of AEC II gene expression profiles which occurred early after T cell recognition of alveolar antigen tended to persist into the chronic phase of inflammation. For example, there was a rapid up-regulation of MHC class II subunit expression, but decreased expression of cytoskeletal genes both early after T cell transfer as well as in AEC II isolated from SPC-HA/TCR-HA mice (Table 1 and Figure 4, 5). However, there were notable exceptions to this pattern, such as was observed with CXCL13 expression, which was clearly down-regulated in AEC II isolated from the chronically inflamed lung of SPC-HA/TCR-HA double transgenic mice but induced acutely in AEC II cells 3 and 6 days after T cell transfer (confirmed by real-time RT-PCR; Figures 5, 6).

**Table 1:** Selected genes differentially expressed in AEC II upon airway inflammation

**Gene (functional category)**	**Symbol**	**SPC-HA/TCR-HA/SPC-HA**		**Fold change**
		**Array1**	**Array2**	**Array1/Array2**
*Genes associated with cell cycle*				

cyclin D2	Ccnd2	208/507	250/648	-2,1/-2,1
transforming growth factor, beta 3	Tgfb3	93/311	87/188	-3,0/-2,2

*Genes associated with cell adhesion*				

procollagen, type IV, alpha 5	Col4a5	89/208	72/224	-1,9/-2,8
procollagen, type XIV, alpha 1	Col14a1	194/2003	142/1858	-9,8/-13,1
fibronectin 1	Fn1	252/2564	407/2813	-9,9/-8,6
dermatopontin	Dpt	250/5627	277/3997	-11,8/-11,6
claudin 18	Cldn18	592/261	1845/445	2,3/3,9

*Genes associated with antigen presentation and processing*				

major histocompatibility complex, class I, B	H2-Q7	1386/85	1666/109	17,6/20,3
major histocompatibility complex, class II, DR alpha	H2-Ea	5720/2661	5207/2187	2,2/2,4
major histocompatibility complex, class II, DQ beta 2	H2-Ab1	2217/1008	3971/1286	2,1/2,9
major histocompatibility complex, class II, DQ alpha 1	H2-Aa	4028/2019	6314/1859	1,9/1,8
major histocompatibility complex, class II, DR beta 1	H2-Eb1	2072/1013	2882/1100	1,9/2,3
major histocompatibility complex, class II, DM alpha	H2-DMa	406/291	961/293	1,6/3,2
proteasome (prosome, macropain) subunit, beta type, 7	Psmb7	418/222	252/117	2,9/2,2
proteasome (prosome, macropain) subunit, beta type, 8	Psmb8	664/223	634/310	2,5/2,2
proteasome (prosome, macropain) subunit, beta type, 9	Psmb9	317/122	528/244	2,8/2,5
beta-2-microglobulin	B2m	8579/4177	8784/3119	2,1/2,9
transporter 1 ATP-binding cassette, sub-family B (MDR/TAP)	Tap1	277/107	283/120	2,4/3,0

*Genes associated with transport*				

potassium inwardly-rectifying channel, subfamily J, member 15	Kcnj15	946/253	1160/231	4,0/4,9
lipocalin 2	Lcn2	11034/3130	13952/1966	3,6/7,4
sodium channel, nonvoltage-gated, type I, alpha polypeptide	Scnn1a	405/292	448/225	2,1/2,4

*Genes associated with immune response*				

Chemokine (C-X-C motif) ligand 1	CXCL1	313/96	235/64	2,5/3,1
Chemokine (C-X-C motif) ligand 13	CXCL13	128/556	100/634	-4,5/-5,9
Chemokine (C-C motif) ligand 12	CXCL12	253/1827	211/1541	-6,7/-7,4
Chemokine (C-X-C motif) ligand 20	CCL20	188/11	141/10	17,1/11,5
chemokine (C-C motif) ligand 11	CCL11	39/302	30/162	-8,5/-4,1

*Genes associated with proteolysis and peptidolysis*				

Matrix metalloproteinase 2	MMP2	154/1788	116/1504	-10,8/-10,3
Matrix metalloproteinase 3	MMP3	51/599	67/547	-10,8/-10,9
Matrix metalloproteinase 23	MMP23	102/685	143/568	-6,2/-3,8
Tissue inhibitor of metalloproteinase 1	TIMP1	54/842	70/569	-11,1/-8,6
Tissue inhibitor of metalloproteinase 2	TIMP2	313/2265	388/2576	-8,6/-8,5
Tissue inhibitor of metalloproteinase 3	TIMP3	623/2935	434/3363	-3,0/-6,0

*Genes associated with cytoskelett*				

elastin	Eln	150/524	177/398	-4,1/-2,5
gelsolin	Gsn	1438/16701	1620/15697	-8,1/-9,7
vimentin	Vim	204/1974	308/2043	-9,6/-6,5
tubulin, alpha 1	Tuba1	1285/6486	1145/6076	-4,7/-5,3

*Genes associated with metabolism*				

vanin 1	Vnn1	1752/200	993/181	9,6/6,3
5,10-methylenetetrahydrofolate reductase	Mthfr	141/300	114/276	-2,0/-2,2
paraoxonase 1	Pon1	460/901	334/774	-2,3/-2,4
hexosaminidase B	Hexb	94/303	93/228	-2,5/-2,2

*Genes associated with signal transduction*				

insulin-like growth factor binding protein 7	Igfbp7	1356/4715	1849/5414	-3,8/-2,52
lipoprotein lipase	Lpl	504/1542	228/1495	-3,3/-5,5
prosaposin	Psap	236/761	319/963	-3,9/-3,0
fibroblast growth factor receptor 3	Fgfr3	160/345	165/304	-2,0/-3,2
interleukin 11 receptor, alpha chain 1	Il11ra1	88/428	146/350	-2,7/-2,7

*Genes associated with signal transduction*				

annexin A1	Anxa11	1230/2328	866/1949	-1,8/-2,4
metallothionein 2	Mt2	118/737	153/758	-5,8/-7,1

*Genes associated with transcription*				

thyrotroph embryonic factor	Tef	350/189	429/203	2,2/2,3
CREBBP/EP300 inhibitory protein 1	Cri1	179/352	116/324	-2,2/-3,1
transcription factor 4	Tcf4	92/462	142/519	-5,0/-4,3
necdin	Ndn	216/1425	152/1895	-5,5/-8,7

*Genes associated with development*				

smoothened homolog (Drosophila)	Smo	148/382	115/335	-2,6/-2,7
four and a half LIM domains 1	Fhl1	704/3478	454/3561	-4,7/-6,4

Differential gene expression was investigated by Affimetrix Gene Chip technology in AEC II from diseased SPC-HA/TCR-HA and healthy SPC-HA mice (n = 3). For each population two independent experiments were performed and data obtained from individual experiments are depicted. The table represents a compilation of regulated genes.

## Discussion

A significant number of lung diseases are presumed to be T cell mediated based in part on the observation of T cell accumulation at sites of disease activity, particularly the interstitial lung diseases (ILD). The ILD represent a broad group of heterogeneous disorders and the participation of CD4^+ ^T cells in various forms of ILD has been suggested. Sarcoidosis, idiopathic interstitial pneumonias, autoimmune connective tissue diseases and pulmonary hemorrhage syndromes represent some of the major categories of ILD. Sarcoidosis, for example, appears to be associated with an exaggerated cellular immune response to an unknown antigen and CD4^+ ^Th1 lymphocytes are important effectors of pulmonary injury in this disease [20,21]. In addition to ILD, it has been postulated that T cells are important contributors in other pulmonary disorders such as chronic obstructive pulmonary disease (COPD) and asthma [22,23]. In these, it is hypothesized that cigarette smoke or allergen induced immune responses can, under certain conditions, progress to T cell mediated autoimmune disease. Recently it has been suggested that smoking-induced emphysema may represent an autoimmune disease of sorts, in which the presence of Th1 responses to a specific lung antigen correlates with emphysema severity [21]. Furthermore, oligoclonal CD4^+ ^T cell expansion has been suggested to contribute to the pathogenesis of obliterative bronchiolitis [24]. Although there is growing evidence that CD4^+ ^T cells contribute to various pulmonary disorders, little is known concerning the role of AEC II cells in T cell mediated lung injury. To expand our understanding of the roles of selected cell types in the induction and progression of inflammatory pulmonary processes, animal models represent tools of extraordinary value. To explore the contribution of AEC II gene expression in T cell mediated lung inflammation, we made use of a transgenic mouse model of chronic T cell mediated lung inflammation that mimics some of the features of the interstitial lung discussed above, and that was previously established [15]. We report here the application of flow cytometry to efficiently isolate alveolar type II epithelial cells from mouse lungs by negative selection followed by whole genome transcriptome analysis. Gene expression profiling has emerged as an important tool in the characterization of complex molecular responses in inflammation and disease. The use of isolated cellular subpopulations has proven to be more informative than whole tissues in dissecting the roles of individual cell types in disease development in general, and immune regulation in particular. Comparative genetic fingerprinting of AEC II isolated from healthy mice and mice suffering from severe lung inflammation promises to be extremely informative regarding the role of AEC II in the induction and regulation of pulmonary immunity and inflammation.

Though confirmation of protein expression is essential, morphological changes in AEC II phenotype and array data suggest very active participation of alveolar epithelial cells in inflammatory processes in the lung. Using Affymetrix GeneChip experiments we identified a heterogeneous set of more than 322 genes differentially expressed in AEC II under pathophysiologic conditions. Variations in signal intensities between experimental repetitions may account for slight differences in the disease progression in individual pooled mice as well as for differences in cRNA synthesis and hybridization efficiencies between two array experiments. To exclude as far as possible that changes in gene expression occur as a consequence of the isolation procedure, care was taken to purify AEC II from the different mouse pools strictly following the described protocol, i.e. avoiding variations of incubation times or temperature, etc. Therefore, the influence of cell isolation procedure on gene expression in AEC II cells from healthy versus inflamed lungs will subtract from each other and account for changes in the molecular signature of AEC II as a consequence of CD4^+ ^T cell mediated lung inflammation.

The differential expression of several immune modulating molecules like TGF-β3 or the various chemokines and chemokine ligands observed, suggests that in an inflamed environment AEC II may interact with resident and mobile neighbour cells via secreted and diffusible signals [9]. Members of the transforming growth factor-beta family are linked to proliferation or secretory activities of AEC II. It has been shown that TGF-β3 production by AEC II is dynamically down-regulated during the proliferative phase of recovery from acute hyperoxic injury [25]. Consistent with this, TGF-β3 expression was down-regulated in AEC II from the inflamed lung, and since AEC II represent the stem cells for alveolar type I epithelial cells (AEC I), this suggests a role of the TGF-β family in AEC II proliferative responses and/or the cellular hypertrophy of AEC II observed in the inflamed lung.

In addition to TGF-β3, the CXC chemokines CXCL2, CXCL13 and CXCL12 were also differentially expressed in AEC II from inflamed compared to healthy lungs (Figure 4, 5, 6, Table 1). These chemokines praticipate in the process of attracting various cell populations into the lung. CXCL12 and CXCL13 bind to CXCR4 and CXCR5, which are primarily expressed on T lymphocytes or on circulating fibrocytes [26]. Interestingly, CXCL12 and CXCL13 expression was induced shortly after T cell recognition of epithelial antigen (Figure 5, 6 and data not shown) and massive lymphocytic infiltrates were observed shortly after T cell transfer (data not shown). Furthermore, down-regulation of T cell chemoattractants was evident at later stages of inflammation (Figure 4 and Table 1) and could contribute to a more controlled infiltration of specific T cells into the lung. Accordingly it has been shown that CXCL13 plays an important role in the development of inducible bronchus associated lymphoid tissue (iBALT) in respiratory immunity [27] by attracting T lymphocytes. It has been suggested that infection or inflammation triggers the organization of lymphoid structures in the lung of both mice and humans [28,29], though this is somewhat controversial. These structures do not fit the classical definition of BALT, as they are not formed independently of antigen [30,31]. Because the iBALT appears in the lung only after infection or inflammation, it is generally assumed that iBALT is simply an accumulation of effector cells that were initially primed in conventional lymphoid organs. The neo-formation of iBALT is caused by inflammatory responses which directly promote the recruitment, priming and expansion of antigen-specific lymphocytes [27]. It is interesting to speculate that AEC II in SPC-HA/TCR-HA double transgenic mice, after the initial inflammatory responses, down-regulate CXCL13 expression in order to counteract new formation of iBALT and infiltration of specific T cells.

The chemokine CXCL2 is involved in attraction of polymorphonuclear granulocytes to sites of infection [32]. These neutrophils play an important role as regulators of immune responses through release of cytokines such as IL-1, IL-3, IL-6, IL-12, tumor necrosis factor-α (TNF-α) or TGF-β as well as chemokines such as CCL2 (MCP-1) or CCL20 (MIP-3α) [33,34].

Elevated expression of CCL20 by AEC II has been shown to attract other pro-inflammatory cells [34,35]. CCL20, which was dramatically up-regulated in the inflamed lung (Figure 4, 5, 6, Table 1), has been shown to be constitutively produced by AEC II cells and can attract immature dendritic cells (imDC) to the lung [36,37]. Immature dendritic cells are known to exert immune modulatory functions and may contribute to the establishment of a controlled immune response in SPC-HA/TCR-HA double transgenic mice. In contrast to CCL20, CCL11 (eotaxin), an eosinophil chemoattractant, was dramatically down-regulated in AEC II from the inflamed lung (Figure 4 and 6, Table 1). Not surprisingly, anti-CCL11 reduced eosinophils infiltration of the lungs of RSV-infected mice. In addition, however, anti-CCL11 also caused inhibited CD4-T-cell influx [38]. Together, these data indicate an active immune regulatory function of AEC II in inflammatory pneumonitis involving the expression and secretion of soluble mediators that may affect other immune cells with regulatory features which may amplify, or interfere with, inflammatory responses in the lung.

Although gene expression data provide evidence that AEC II may (either directly or indirectly) exhibit immune regulatory functions, we also identified genes involved in the induction of T cell mediated immunity. In this context it is interesting to note that the expression levels for molecules involved in antigen processing and presentation were up-regulated in AEC II obtained from diseased mice. For instance, increased expression of molecules needed for the MHC class-II restricted antigen presentation, like H2-Ea and H2-Ab1, but also invariant chain (CD74), was observed. Furthermore, expression of genes encoding for the transporter associated with antigen processing (TAP1) and various proteasomal subunits, all related with MHC class I presentation, were increased (Figure 4, 5, 6, Table 1). This effect was observed both in SPC-HA/TCR-HA mice that exhibit chronic inflammation as well as in AEC II from SPC-HA mice shortly after T cell transfer. Up-regulation of MHC encoded genes is likely the result of interferon (IFN)-γ production by the CD4^+ ^T cells, and is well known to induce the transcription of genes encoded within the MHC region. Based on our previous observation in an adoptive transfer model for CD8^+ ^T cell mediated pulmonary inflammation, as well as in cell culture experiments [13,14,39], we have strong evidence that T cell antigen recognition triggers inflammatory gene expression in AEC II cells, a significant portion of which is IFN-γ dependent. Although CD4^+ ^T cell derived IL-2 and IFN-γ are likely pro-inflammatory mediators that trigger AEC II gene expression in SPC-HA/TCR-HA mice or SPC-HA mice after adoptive T cell transfer, it is possible that other T cell derived factors contribute to the observed changes in AEC II gene expression, such as TNF-α.

Further genes differentially expressed in AEC II upon airway inflammation are cyclin A2 and cyclin D2, both involved in cell cycle regulation [40,41] and several matrix metalloproteinases (MMP) and tissue inhibitor metalloproteinases (TIMP), all of which are critical in repair and remodelling in response to injury [42,43]. In addition to these, genes with roles in adhesion, cytoskeletal function, transport, metabolism, signal transduction, transcription and development suggest that AEC II are active participants in all aspects of immune regulation, inflammation and responses to injury. The impact of these gene products on the ethiopathogenesis of pulmonary inflammation remain to be elucidated in further detail.

## Conclusion

We have developed a new AEC II isolation protocol based on flow cytometric negative selection for the isolation of cell populations of high purity and viability. Employing this technique, we determined the genome-wide profile of gene expression in response to T cell-mediated interstitial pneumonitis. Overall, these results provide a detailed description of AEC II gene expression under pathophysiologic, autoimmune conditions. Differentially expressed genes of diverse molecular functions have been identified that may be critical for numerous physiologic activities, some of which may be currently unappreciated. Data obtained by such analysis will help to understand the function of these important immune cells in the respiratory system and may point out strategies for intervention in the progression of chronic inflammatory processes in the lung.

## Competing interests

The author(s) declare that they have no competing interests.

## Authors' contribution

MG carried out all experiments except for immunofluorescence stainings, was involved in the interpretation of data, designed figures and tables. LG performed cell sorting. SP was involved in mice genotyping and assisted with most of the experiments. MK performed immunoflurescence staining and interpreted this set of data. SD and ADG performed histological examination and scoring. RIE provided basic protocols, contributed to the conception of the study and critically revised the manuscript. JB has substantially contributed to the overall study design and also revised the manuscript. DB is primary investigator, who conceived the study, helped to prepare figures and wrote the manuscript. All authors have read and approved the final manuscript.
